# A System of Practical Surgery

**Published:** 1904-09

**Authors:** 


### BOOK REVIEWS.

---

A SYSTEM OF PRACTICAL SURGERY. By Prof. E. von Bergmann, M. D., Berlin, Prof. P. Bruns, M. D., Tübengen, and Prof. J. von Mikulicz, M. D., Breslau. Translated and Edited by Wm. T. Bull, M. D., Columbia University, Etc., New York, and John B. Solly, M. D. Vol. III. Surgery of the Extremeties.

This volume covers very completely the malformations, diseases, injuries, etc., of not only the extremities, but of the shoulder and hip. The same good illustrations (save many of the Fray pictures) are found in this and Volume IV. As in the preceding volumes, instruments and procedures are clearly illustrated and the text is practical and interesting. It would seem that even the rarest of abnormalities, diseases or injuries are included and the treatment described.

Vol. IV. is entitled Surgery of the Alimentary Tract, but it also treats of conditions of the abdominal wall, and all the abdominal viscera. Appendicitis is most thoroughly discussed with profuse illustrations, some being beautiful plates. Gastric and intestinal surgery also receives full mention and illustration, as do the hernias. The malignant growths are not so fully treated of in any of the volumes.

